# Control of entanglement dynamics in a system of three coupled quantum oscillators

**DOI:** 10.1038/s41598-017-09989-2

**Published:** 2017-08-30

**Authors:** J. C. Gonzalez-Henao, E. Pugliese, S. Euzzor, R. Meucci, J. A. Roversi, F. T. Arecchi

**Affiliations:** 1 Instituto de Física “Gleb Wataghin”, Universidade Estadual de Campinas, Unicamp, 13083-970 Campinas, São Paulo Brazil; 2 Departamento de Física, Universidad de Sucre, Cra 28 No 5-267 Puerta Roja, Sincelejo, Sucre Colombia; 3 Istituto Nazionale di Ottica-CNR Largo E. Fermi 6, 50125 Firenze, Italy; 4 Emeritus of Physics, Università degli Studi di Firenze, Firenze, Italy

## Abstract

Dynamical control of entanglement and its connection with the classical concept of instability is an intriguing matter which deserves accurate investigation for its important role in information processing, cryptography and quantum computing. Here we consider a tripartite quantum system made of three coupled quantum parametric oscillators in equilibrium with a common heat bath. The introduced parametrization consists of a pulse train with adjustable amplitude and duty cycle representing a more general case for the perturbation. From the experimental observation of the instability in the classical system we are able to predict the parameter values for which the entangled states exist. A different amount of entanglement and different onset times emerge when comparing two and three quantum oscillators. The system and the parametrization considered here open new perspectives for manipulating quantum features at high temperatures.

## Introduction

In the last decades, the peculiar features of quantum mechanics have been exploited to introduce promising new faster calculation and process information methodologies. Since in these new approaches the major role is played by entanglement, many efforts, at both the theoretical and experimental level, have been performed to better understand and control such a quantum peculiarity. In particular, the effects of decoherence in open quantum systems at high temperature have been studied. Indeed, the interaction with a hot environment determines in general the disappearance of quantum behaviour^1, 2^. Several schemes have been proposed to overcome or, at least, to reduce the influence of temperature and of the environment in the losses of coherence. Duan and Guo^1^ proposed to pair each qubit with an ancilla qubit and to encode the states of the system into states of qubit pairs. In the same direction, Gonzalez-Henao and Roversi^3^ proposed a third qubit to protect entanglement in a two qubit system at high temperature. More recently, Yu and Ye^4^ exploited the weak measurement to protect entanglement under decoherence in non-inertial frames.

Entanglement in coupled oscillators with variable frequency have been considered in refs 5, 6. The relationship between the dynamic instabilities and entanglement in a system of two quantum parametric oscillators has been investigated offering the way to obtain the survival of quantum features even at high temperature^7, 8^.

Here we use an external pulse to control the dynamic instabilities in a system of three coupled oscillators. By the close relation between the dynamic instability and entanglement the bipartite entanglement of the system at high temperature regime can be controlled. The control pulse is the propeller of the dynamic instabilities driving the quantum system towards or away from entangled states. This feature allows to maintain the entanglement state for a long time. This method may open a way for manipulating quantum features at high temperature regimes.

## The Quantum System

We consider a system of two and three coupled quantum parametric oscillators in equilibrium with a common heat bath at temperature *T*. The system is described by the following Hamiltonian

1 $${H}_{T}={H}_{S}+{H}_{SR}$$

2 $${H}_{S}=\frac{1}{2}\sum _{i=1}^{N}\,[\frac{{P}_{i}^{2}}{{m}_{i}}+{m}_{i}{\omega }^{2}(t){X}_{i}^{2}]+\sum _{i > j=1}^{N}c(t){X}_{i}{X}_{j}$$

3 $$\begin{array}{c}{H}_{SR}={\sum }_{k=1}^{{\rm{\infty }}}\,[\frac{{p}_{k}^{2}}{2{m}_{k}}+\frac{{m}_{k}{\omega }_{k}^{2}{x}_{k}^{2}}{2}-\sqrt{2}{c}_{k}{x}_{k}({\sum }_{i=1}^{N}{X}_{i})+\frac{{c}_{k}^{2}}{2{m}_{k}{\omega }_{k}^{2}}{({\sum }_{i=1}^{N}{X}_{i})}^{2}]\end{array}$$

The total Hamiltonian *H*_*T*_ consists of *H*_*S*_ that represents a system of oscillators with *N* = 2, 3 (with equal mass *m*_i_ = *m*_0_) and their interactions and *H*_*SR*_ that represents the interaction between oscillators and thermal reservoir. *ω*(*t*) is the angular frequency and {*X*_i_, *P*_i_} the position and momentum operators for the oscillators with *i* = 1, 2, 3; similarly *ω*_*k*_ and {*x*_*k*_, *p*_*k*_} characterize the environment oscillators. The oscillators 1, 2 and 3 are coupled to each other by the functions *c*(*t*) and connected to the environment through the constants *c*_*k*_.

In the case *N* = 3 of three coupled oscillators, starting from [*X*] = (*X*_1_, *X*_2_, *X*_3_)^*T*^ and [*P*] = (*P*_1_, *P*_2_, *P*_3_)^*T*^ we can define two new vectors [*X*] = *R*.[*X*′] and [*P*] = *R*.[*P*′] employing the orthogonal transformation *R* which decouples the Hamiltonian *H*_*S*_

4 $$R=(\begin{array}{ccc}\frac{1}{\sqrt{3}} & 0 & -\sqrt{\frac{2}{3}}\\ \frac{1}{\sqrt{3}} & \frac{1}{\sqrt{2}} & \frac{1}{\sqrt{6}}\\ \frac{1}{\sqrt{3}} & -\frac{1}{\sqrt{2}} & \frac{1}{\sqrt{6}}\end{array})$$

In order to simplify the Hamiltonian we introduce the normalized operators *H*_*T*_ → *H*_*T*_/$\hslash$ *ω*_0_, ${X}_{i}\to {X}_{i}\sqrt{\hslash /{m}_{i}{\omega }_{0}}$ and ${P}_{i}\to {P}_{i}\sqrt{{m}_{i}{\omega }_{0}\hslash }$ where *ω*_0_ is the natural frequency of the oscillators 1, 2 and 3. Equivalent transformations are introduced for the operators *x*_*i*_ and *p*_*i*_. The Hamiltonian *H*_*T*_ becomes

5 $${H}_{T}={H}_{1}^{^{\prime} }+{H}_{2}^{^{\prime} }+{H}_{3}^{^{\prime} }$$

6 $$\begin{array}{c}{H}_{1}^{^{\prime} }=\frac{1}{2}[{{\rm{\Omega }}}_{+}^{2}(t){X}_{1}^{{}^{^{\prime} }2}+{P}_{1}^{{}^{^{\prime} }2}]+{\sum }_{k=1}^{{\rm{\infty }}}(\frac{{p}_{k}^{2}}{2}+\frac{{\omega }_{k}^{2}{x}_{k}^{2}}{2}-\sqrt{2}{c}_{k}{x}_{k}{X}_{1}^{^{\prime} }+\frac{{c}_{k}^{2}}{2{\omega }_{k}^{2}}{X}_{1}^{^{\prime} 2})\end{array}$$

7 $${H}_{2}^{^{\prime} }=\frac{1}{2}[{{\rm{\Omega }}}_{-}^{2}(t){X}_{2}^{^{\prime} 2}+{P}_{2}^{^{\prime} 2}]$$

8 $${H}_{3}^{^{\prime} }=\frac{1}{2}[{{\rm{\Omega }}}_{-}^{2}(t){X}_{3}^{^{\prime} 2}+{P}_{3}^{^{\prime} 2}]$$

In the previous equations the new pulsation ${{\rm{\Omega }}}_{+}^{2}(t)=[\omega {(t)}^{2}+2c(t)/{m}_{0}]/{\omega }_{0}^{2}$ and ${{\rm{\Omega }}}_{-}^{2}(t)=[\omega {(t)}^{2}-c(t)/{m}_{0}]/{\omega }_{0}^{2}$ take into account both original angular frequencies and coupling term. The orthogonality of the transformation guarantees that the primed Hamiltonians satisfy (see Supplementary): $[{H}_{1}^{^{\prime} },{H}_{2}^{^{\prime} }]=[{H}_{1}^{^{\prime} },{H}_{3}^{^{\prime} }]=[{H}_{3}^{^{\prime} },{H}_{2}^{^{\prime} }]=0$.

In the case *N* = 2, the *R* matrix is:

9 $$R=(\begin{array}{cc}\frac{1}{\sqrt{2}} & \frac{1}{\sqrt{2}}\\ \frac{1}{\sqrt{2}} & -\frac{1}{\sqrt{2}}\end{array})$$

and the Hamiltonian *H*_*T*_ is decoupled as

10 $${H}_{T}={H}_{1}^{^{\prime} }+{H}_{2}^{^{\prime} }$$

11 $$\begin{array}{ccc}{H}_{1}^{{}^{^{\prime} }} & = & \frac{1}{2}[{{\rm{\Omega }}}_{+}^{2}(t){X}_{1}^{{}^{^{\prime} }2}+{P}_{1}^{{}^{^{\prime} }2}]+{\sum }_{k=1}^{{\rm{\infty }}}(\frac{{p}_{k}^{2}}{2}+\frac{{\omega }_{k}^{2}{x}_{k}^{2}}{2}-\sqrt{2}{c}_{k}{x}_{k}{X}_{1}^{{}^{^{\prime} }}+\frac{{c}_{k}^{2}}{2{\omega }_{k}^{2}}{X}_{1}^{{}^{^{\prime} }2})\end{array}$$

12 $${H}_{2}^{^{\prime} }=\frac{1}{2}[{{\rm{\Omega }}}_{-}^{2}(t){X}_{2}^{^{\prime} 2}+{P}_{2}^{^{\prime} 2}]$$

with ${{\rm{\Omega }}}_{\pm }^{2}(t)=[\omega {(t)}^{2}\pm c(t)/{m}_{0}]/{\omega }_{0}^{2}$, in this case the commutator between the primed Hamiltonians is zero due to the orthogonality of the *R* transformation.

Here we consider, as initial condition, the coherent state $\hat{\rho }(0)=|{\rm{\Psi }}\rangle \langle {\rm{\Psi }}|$ where $|{\rm{\Psi }}\rangle =|{\alpha }_{1}\mathrm{.}.{\alpha }_{N}\rangle$ is a tensorial product of coherent states associated with parametric oscillators (see Supplementary).

The coherent character of the initial Gaussian state is preserved by orthonormal transformations originated from *R* and it is maintained during the time evolution thanks to the bilinearity in the coordinates of the Hamiltonians (5) and (10). This implies that every quantum correlations will be obtained from the Covariance Matrix (CM) whose elements are given by

13 $${\sigma }_{{ {\mathcal R} }_{i}{ {\mathcal R} }_{j}}=\frac{1}{2}\langle { {\mathcal R} }_{i}{ {\mathcal R} }_{j}+{ {\mathcal R} }_{j}{ {\mathcal R} }_{i}\rangle -\langle { {\mathcal R} }_{i}\rangle \langle { {\mathcal R} }_{j}\rangle $$

with $[ {\mathcal R} ]=({X}_{1},{P}_{1},{X}_{2},{P}_{2},{X}_{3},{P}_{3})$. The elements of the Covariance Matrix are easily obtained by using the primed operators and since the primed Hamiltonians commute between themselves an independent analysis leads to ${\sigma }_{{X}_{i}^{^{\prime} }{X}_{j}^{^{\prime} }}={\sigma }_{{P}_{i}^{^{\prime} }{P}_{j}^{^{\prime} }}=0$ for *i* ≠ *j* (see Supplementary).

In the calculation of the CM for the Hamiltonian (6) and (11) we have used the path integral formulation of Feynman where we calculate the propagator of the system *J* (see Supplementary and refs 9, 10), the temporal evolution of the density matrix is

14 $$\begin{array}{rcl}\rho ({\mathscr{X}},{\mathscr{Y}},t) & = & \int d{\mathscr{X}}^{\prime} d{\mathscr{Y}}^{\prime} J({\mathscr{X}},{\mathscr{Y}},{\mathscr{X}}^{\prime} ,{\mathscr{Y}}^{\prime} ,t)\rho ({\mathscr{X}}^{\prime} ,{\mathscr{Y}}^{\prime} \mathrm{,0)}\\ \rho ({\mathscr{X}}^{\prime} ,{\mathscr{Y}}^{\prime} \mathrm{,0)} & = & \langle {\mathscr{X}}^{\prime} |\hat{\rho }\mathrm{(0)}|{\mathscr{Y}}^{\prime} \rangle =\langle {\mathscr{X}}^{\prime} |{\rm{\Psi }}\rangle \langle {\rm{\Psi }}|{\mathscr{Y}}^{\prime} \rangle \end{array}$$

To calculate the CM elements for the Hamiltonians (7), (8) and (12) we have used the Heisenberg representation (see Supplementary).

## Experimental

In previous works^7, 8^, it has been shown that the onset of the entanglement is connected to the diverging solutions of the CM element equations of the operators *X*′ and *P*′. We indicated with ${{\mathbb{X}}}_{+}(t)$ and ${{\mathbb{X}}}_{-}(t)$ the time dependence of the CM elements, in both cases of *N* = 2 and *N* = 3, for the oscillators with pulsation Ω_+_ and Ω_−_ respectively. ${{\mathbb{X}}}_{+}(t)$ and ${{\mathbb{X}}}_{-}(t)$ evolve in time according to the following differential equations

15 $${\ddot{{\mathbb{X}}}}_{-}(t)+{{\rm{\Omega }}}_{-}^{2}(t){{\mathbb{X}}}_{-}(t)=\mathrm{0,}$$

16 $${\ddot{{\mathbb{X}}}}_{+}(t)+[{{\rm{\Omega }}}_{+}^{2}(t)-\frac{{\gamma }^{2}}{4}]{{\mathbb{X}}}_{+}(t)=0.$$

where *γ* is the dissipation rate of the reservoir.

We found that only the dynamical behaviour of the oscillators characterized by Ω_−_ determines the survival of the entanglement at high temperature. Then we focused our attention on equation (15) to describe, also in experimental way, the space parameter regions where we expect entanglement arises.

We specialised the discussion to the case $c(t)=c{m}_{0}{\omega }_{o}^{2}$ and $\omega {(t)}^{2}={\omega }_{o}^{2}[1+A\mathrm{(1}+mf({\omega }_{p},t))cos({\omega }_{d}t)]$, with *f*(*ω*_*p*_, *t*) a suitable function and *ω*_*d*_ the external driving pulsation. In that way we can define the dimensionless pulsation ${{\rm{\Omega }}}_{-}^{2}=[\omega {(t)}^{2}-c(t)/{m}_{0}]/{\omega }_{0}^{2}=\omega {(t)}^{2}/{\omega }_{o}^{2}-c={\omega }_{r}^{2}+A[1+mf({\omega }_{p},t)]cos({\omega }_{d}t)$, with ${\omega }_{r}^{2}=1-c$.

**Figure 1:**
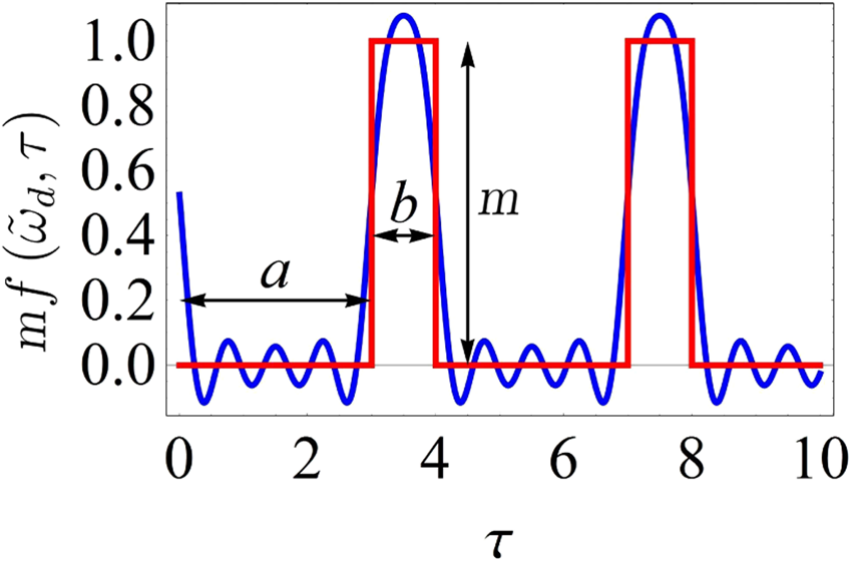
Schematic representation of the external perturbation $mf({\tilde{\omega }}_{p},\tau )$. The blue curve is the result of superposition of the first five Fourier harmonics (*a* = 3 and *b* = 1); the red step function is the experimental stimulus introduced into the Mathieu’s oscillator.

The experimental tests have been performed implementing an analog electronic version of the equation (15) with the Ω_−_ introduced above, which corresponds to a perturbed Mathieu oscillator (see section Methods and Fig. 6). In terms of first order differential equations the system is described by

17 $$\{\begin{array}{c}\dot{x}=y\\ \dot{y}=-\{{\omega }_{r}^{2}+A[1+mf({\mathop{\omega }\limits^{ \sim }}_{p},\tau )]cos({\mathop{\omega }\limits^{ \sim }}_{d}\tau )\}x\end{array}$$

where we assume ${\tilde{\omega }}_{d}={\omega }_{d}/{\omega }_{o}$, ${\tilde{\omega }}_{p}={\omega }_{p}/{\omega }_{o}$ and *τ* = *tω*_*o*_, with *ω*_*o*_ related to the ground state energy of quantum oscillators. The term $m\cdot f({\tilde{\omega }}_{p},\tau )$ represents the analytic form of the external stimulus and consists of a pulse train with adjustable amplitude and duty cycle (see blue curve in Fig. 1).

From a mathematical point of view the function *f* can be obtained as superposition of Fourier harmonics of angular frequency ${\tilde{\omega }}_{p}=2\pi /(a+b)$, where *a* + *b* is the dimensionless perturbation period. It can be shown that the stability maps are almost invariant if more than five harmonics are used. This allows us to experimentally use a step function (red curve in Fig. 1) as the external stimulus without affecting the comparison between simulations and experimental data.

**Figure 2:**
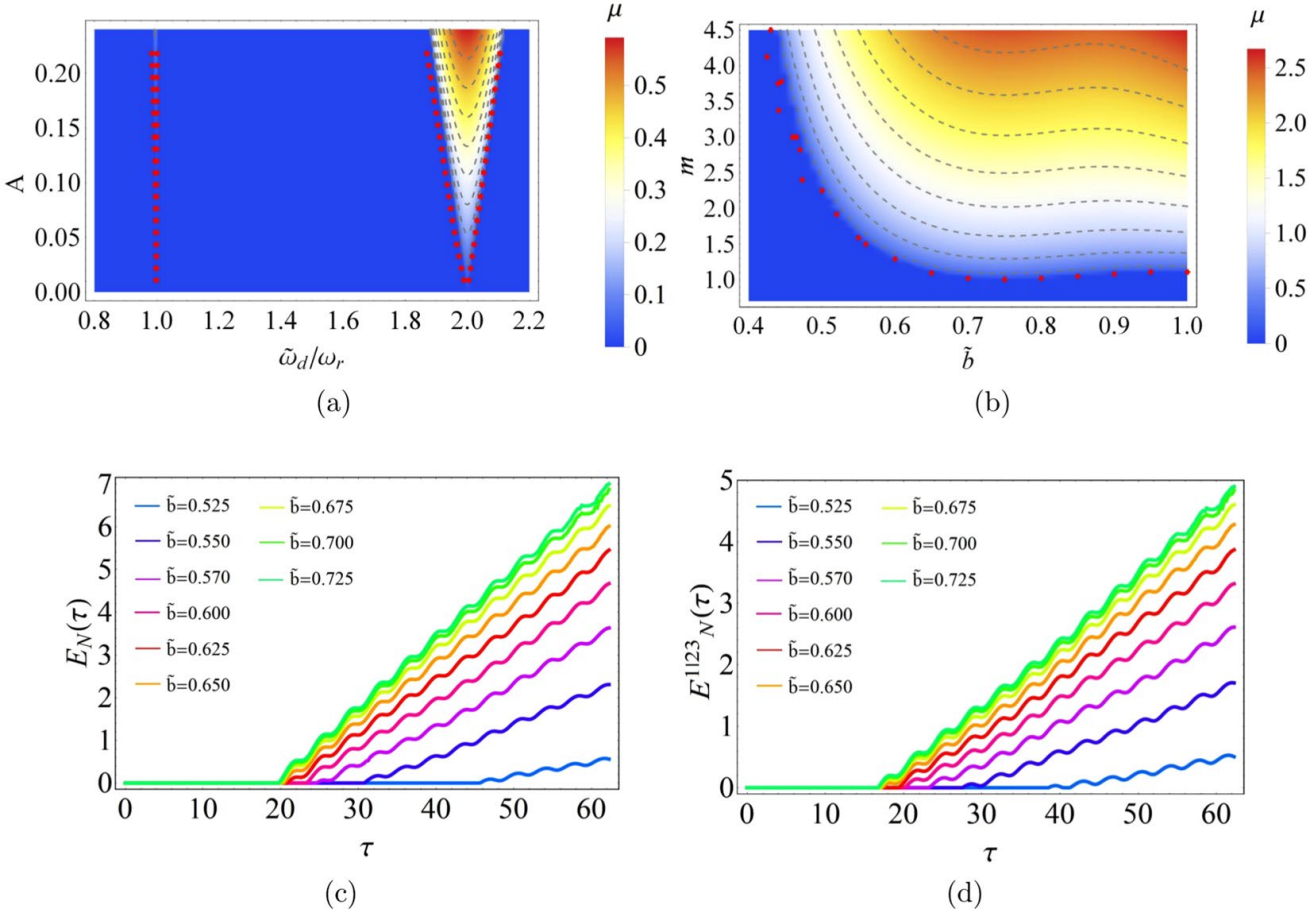
Stability maps and logarithmic negativity. (**a**) Simulated stability map *A* vs ${\tilde{\omega }}_{d}/{\omega }_{r}$ of the dynamical system (17) and superimposed experimental data (red points) for *m* = 0; (**b**) Simulated stability map *m* vs $\tilde{b}$ for the (17), assuming ${\tilde{\omega }}_{d}/{\omega }_{r}=1.7$ and *A* = 0.215, and superimposed experimental data (red points). In both figures the red points delimit the stable from the unstable regions in the experiment. The color bars are associated with the real part *μ* of the Floquet coefficient. (**c**,**d**) Logarithmic negativity *E*_*N*_ vs *τ*, for *N* = 2 and *N* = 3 oscillators respectively (${\tilde{\omega }}_{d}/{\omega }_{r}=1.7$, *A* = 0.215 and *m* = 2.0).

By observing the signal saturation, due to the integrated electronic component limits, we can experimentally reconstruct, in the parameter space ${\tilde{\omega }}_{d}-A$, the instability regions corresponding to ${\tilde{\omega }}_{d}/{\omega }_{r}=1$ and 2 (see Fig. 2(a)). Focusing on ${\tilde{\omega }}_{d}/{\omega }_{r}=2$, where the driving pulsation *ω*_*d*_ approaches twice *ω*_*r*_, the system shows a decreasing amplitude modulation at the angular frequency difference *ω*_*d*_ − *ω*_*r*_, at the same time the signals become increasingly clipped to the saturation values. Corresponding to two couples of fixed values $({\tilde{\omega }}_{d}/{\omega }_{r},A)=(2,0.215)$ and (1.7, 0.215) the perturbation $m\cdot f({\tilde{\omega }}_{p},\tau )$ is switched on. The dynamical behaviour of the system is controlled by the control parameter *m* and $\tilde{b}=b/(a+b)$ and the classical instability regions are detected. The stability map in the plane $\tilde{b}-m$ for ${\tilde{\omega }}_{d}/{\omega }_{r}=1.7$ is shown in Fig. 2(b). The experimental points (red dots in Fig. 2) were overlaid to the numerical simulations of instability regions characterized by positive values of the real part *μ* of the so called Floquet coefficient^11^. From a general point of view, Eq. (17) is a periodical and linear differential equation. The Floquet theorem guarantees that, under these conditions, the solution is of the form (*x*(*t*), *y*(*t*))^*T*^ = exp(*Kt*)*F*(*t*)*C*. The two eigenvalues *λ*_±_ of *K* are known as Floquet coefficients. Defining *μ*_±_ = *Re*{*λ*_±_}, the system is unstable when *μ*_+_ = *μ* > 0 as in our case *μ*_−_ = −*μ*_+_.

## Entanglement

We show the difference between the bipartite quantum entanglement in systems of 2 and 3 oscillators. Since the final system state is Gaussian, the theorem of positive partial transpose (*PPT*)^12, 13^ is used as a criterion for entanglement. Furthermore, the logarithmic negativity^12^ is employed to evaluate the entanglement level in the system of two oscillators:

18 $${E}_{N}=\{\begin{array}{cc}0 & if\,{v}_{-}\ge 1/2\\ -\mathrm{log}2{v}_{-} & if\,{v}_{-} < 1/2\end{array},$$

where *v*_−_ is given by:

19 $$2{v}_{-}^{2}={I}_{1}+{I}_{2}-2{I}_{3}-\sqrt{{({I}_{1}+{I}_{2}-2{I}_{3})}^{2}-2{I}_{4}},$$

with *I*_1_ = det[*σ*_11_], *I*_2_ = det[*σ*_22_], *I*_3_ = det[*σ*_12_] and *I*_4_ = det[*σ*], the matrices *σ*_11_, *σ*_22_ and *σ*_12_ being sub-blocks of the covariance matrix

20 $$\sigma =(\begin{array}{cc}{\sigma }_{11} & {\sigma }_{12}\\ {\sigma }_{12}^{T} & {\sigma }_{22}\end{array}).$$

The two oscillators are entangled or separable for *E*_*N*_ ≠ 0 and *E*_*N*_ = 0 respectively. In the case of 3 oscillators we used again the PPT theorem to analise the entanglement bipartite between the oscillator 1 with the oscillators 2 and 3. As the hamiltonian (1) is invariant to coordinate changes the CM is fully symmetric^14^, then we can use the bi-partite logarithmic negativity given by

21 $${E}_{N}^{1| 23}=\{\begin{array}{cc}0 & if\,{\mathop{n}\limits^{ \sim }}_{-}\ge 1\\ -\mathrm{log}{\mathop{n}\limits^{ \sim }}_{-} & if\,{\mathop{n}\limits^{ \sim }}_{-} < 1\end{array}$$

where ${\tilde{n}}_{-}$ is a eigenvalue of the CM transpose (only one of the 6 eigenvalues of the *σ* transpose can be negative) of the system with 3 oscillators^14^ (see Supplementary).

## Results

In all calculations the function *f*(*ω*, *τ*) has been approximated by the first five terms of its Fourier series and we used the following values *A* = 0.215, ${\tilde{\omega }}_{d}/{\omega }_{r}=1.7$, *m* = 2, *c* = 0.0591, temperature of the reservoir $\tilde{T}={K}_{B}T/\hslash {\omega }_{0}=100$ and dissipation rate *γ* = 0.01*ω*_0_. The initial condition of the system with 2 oscillators is the coherent state $|{\rm{\Psi }}\rangle =|\sqrt{2}\alpha 0\rangle$ that in the primed coordinates becomes $|{\rm{\Psi }}\rangle =|\alpha \alpha \rangle$. The initial condition of the system with 3 oscillators is the coherent state $|{\rm{\Psi }}\rangle =|{\alpha }_{1}{\alpha }_{2}{\alpha }_{3}\rangle$ ${\alpha }_{1}=-\alpha (\sqrt{2}-1)/\sqrt{3}$, ${\alpha }_{2}=\alpha (3\sqrt{2}+2\sqrt{3}+\sqrt{6})\mathrm{/6}$ and ${\alpha }_{3}=\alpha (-3\sqrt{2}+2\sqrt{3}+\sqrt{6})\mathrm{/6}$. In the primed coordinates this state is expressed by $|{\rm{\Psi }}\rangle =|\alpha \alpha \alpha \rangle$ with $\alpha =\mathrm{1/}\sqrt{2}$.

In Fig. 2(c) we report, for *N* = 2, the logarithmic negativity against the dimensionless time *τ*, for different values of the duty cycle $\tilde{b}$. For values of $\tilde{b}$ for which *μ* = 0 (see Fig. 2(b)) the system does not present entanglement. Otherwise, for $0.515\le \tilde{b}\le 1.000$, where *μ* > 0 and after a certain time the system presents entanglement. Once the system has acquired entanglement this grows approximately linearly with small superimposed oscillations. Figure 2(d) shows the bipartite logarithmic negativity ${E}_{N}^{\mathrm{1|23}}$ for three oscillators with focus on composition 1 with the couple 2 and 3. Because CM is symmetric, any subdivision of the system leads to the same degree of entanglement. In other words ${E}_{N}^{\mathrm{1|23}}={E}_{N}^{\mathrm{2|31}}={E}_{N}^{\mathrm{3|12}}$, as verified. In Fig. 2(d), we observe that for the same values of $\tilde{b}$ and *μ* > 0 where entanglement is detected for two oscillators, a similar behaviour emerges for three oscillators with the difference that the function ${E}_{N}^{\mathrm{1|23}}(\tau ) < {E}_{N}(\tau )$. This reduction of entanglement, due to the third oscillator, is consistent with the monogamy inequality conjectured by Coffman, Kundu and Wootters^15^ and extended by Osborne and Verstraete^16^. The same level of entanglement in the two cases (system with two oscillators and system with three oscillators) is reached if and only if for the three oscillators, the entanglement between the oscillators 2 and 3 is zero, i.e. 2 and 3 are separable oscillators.

**Figure 3:**
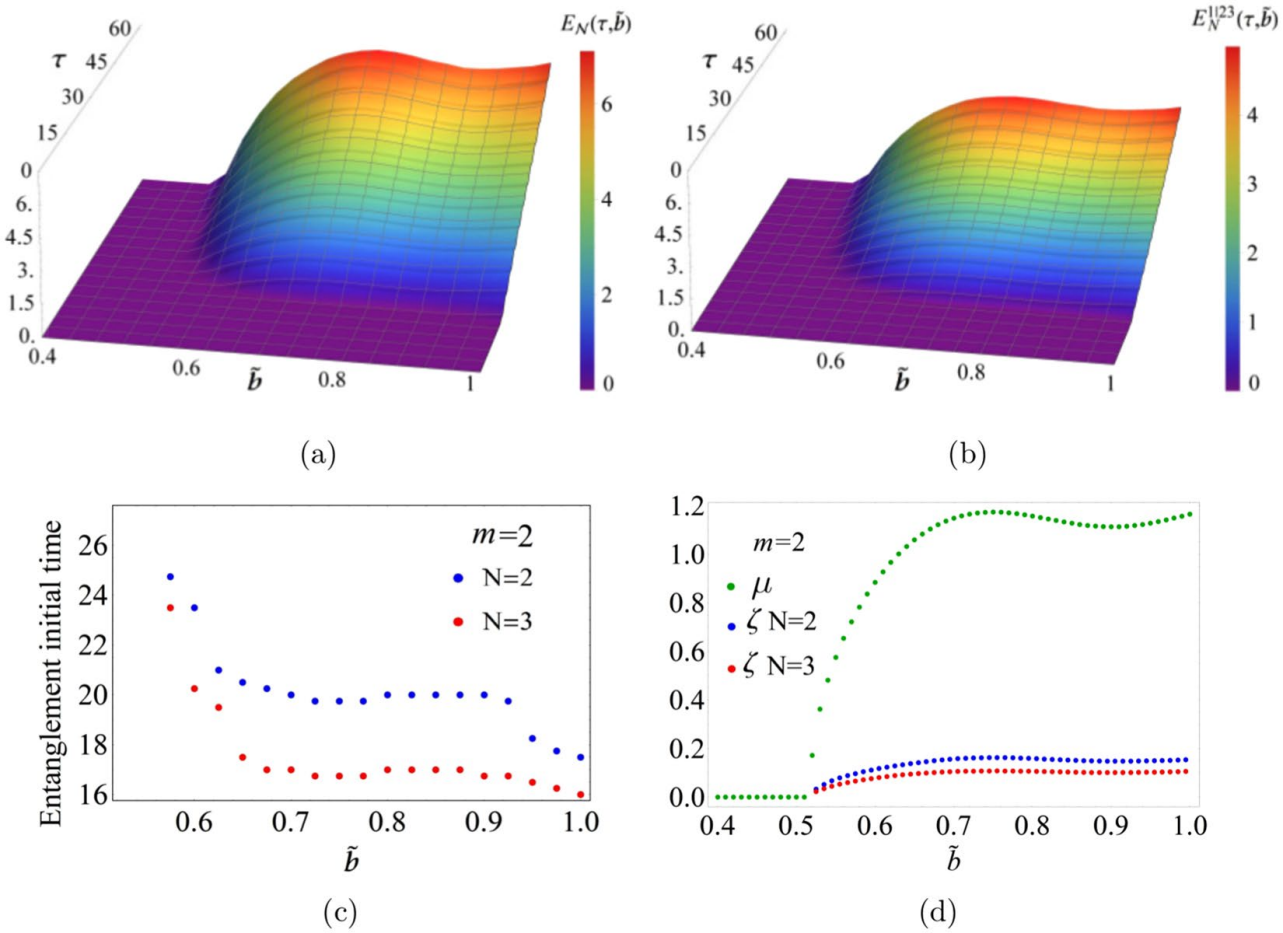
Bipartite entanglement. (**a**), (**b**) Plots of the bipartite entanglement as a function of time *τ* and $\tilde{b}$ (${\tilde{\omega }}_{d}/{\omega }_{r}=1.7$, *A* = 0.215 and *m* = 2.0) for the system with 2 and 3 oscillators respectively. (**c**) Initial entanglement time for two (blue curve) and three (red curve) oscillators. (**d**) Real part *μ* of Floquet coefficient (green curve) and entanglement rate *ζ* for 2 (blue curve) and 3 (red curve) oscillators as function of $\tilde{b}$.

In Fig. 3(a) and (b) the surfaces of the bipartite logarithmic negativity ${E}_{N}(\tau ,\tilde{b})$ and ${E}_{N}^{\mathrm{1|23}}(\tau ,\tilde{b})$ respectively, generated from Fig. 2(c) and (d) are plotted as function of *τ* and $\tilde{b}$. These surfaces highlight the different value of the maximum entanglement (*E*_*N*_ ≈ 7.01 and ${E}_{N}^{\mathrm{1|23}}\approx 5.03$) between *E*_*N*_ and ${E}_{N}^{\mathrm{1|23}}$. Figure 3(c) reports the initial entanglement time against $\tilde{b}$ for 2 (blue dots) and 3 (red dots) oscillators showing that entanglement occurs first in the latter case.

In Fig. 3(d), the mean entanglement rates *ζ* for 2 and 3 oscillators, corresponding to the surfaces of Fig. 3(a) and (b), and the real part *μ* of the Floquet coefficient (green dots) are plotted as a function of $\tilde{b}$. As already observed in Fig. 2(b–d), the entanglement is different from zero where the classical oscillator is unstable. Furthermore, *ζ* is higher for 2 oscillators, in fact the three oscillators system is faster to develop entanglement but with a lower value.

**Figure 4:**
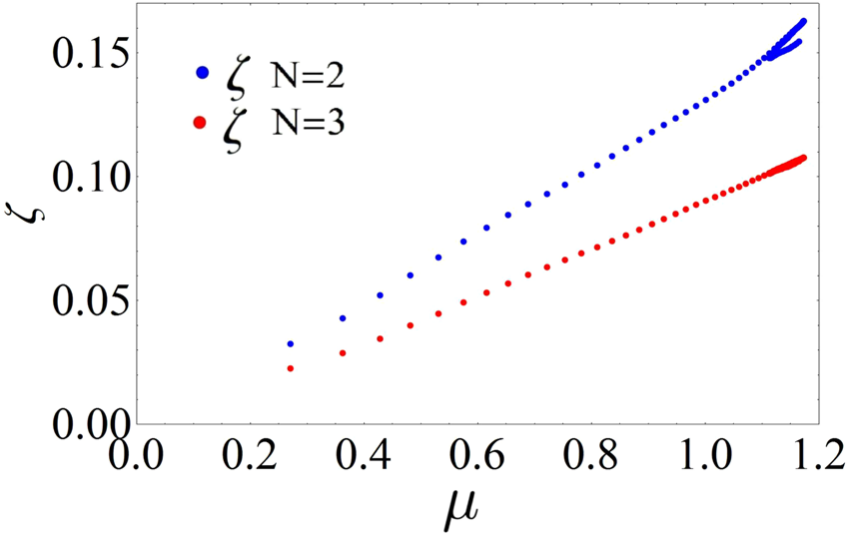
Mean entanglement rate. Mean entanglement rate *ζ* versus the real part *μ* of the Floquet coefficient.

In Fig. 4 we show the average entanglement rate *ζ* as function of *μ* for the two systems. The parameter *ζ* has been numerically obtained by fitting the curves of Fig. 2(c) and (d) using the nonlinear function $E(\tau )={E}_{0}+\zeta \tau +{E}_{m}\,\sin (\bar{\omega }\tau )$.

From this figure it can be noted that the behaviour of *ζ* versus *μ* is linear and that the rate for 2 oscillators is larger than for 3 oscillators. In addition, the relation between *μ* and *ζ* is not bijective since in the system of 2 oscillators there are regions with two values of *ζ* (bifurcation of the entanglement rate) for the same value of *μ*. As it is possible to see in Fig. 3(d), the real part of the Floquet coefficient displays a local minimum as function of $\tilde{b}$ originating the bifurcation in the *ζ* vs *μ* curve.

**Figure 5:**
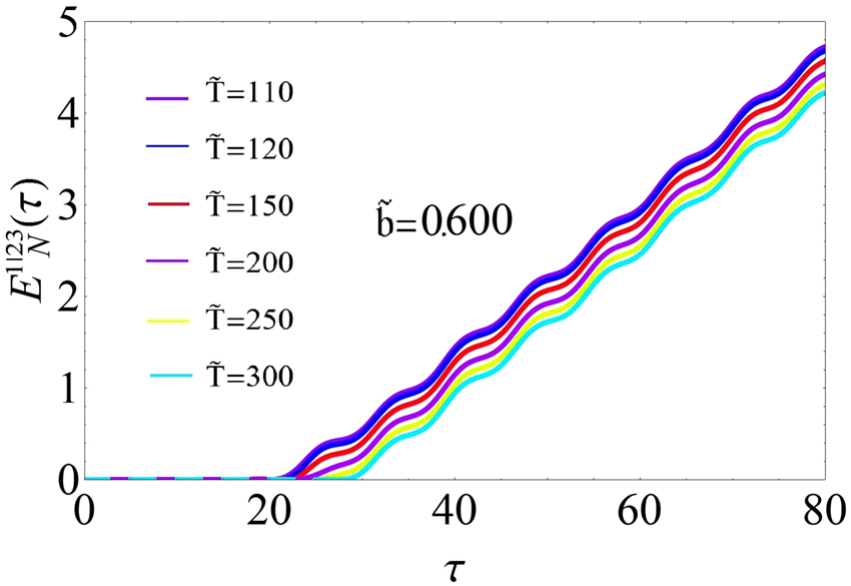
Temperature dependence of the bipartite entanglement. Plots of the bipartite entanglement ${E}_{N}^{\mathrm{1|23}}$ as a function of time *τ* for different values of $\tilde{T}$ for the system with 3 oscillators (${\tilde{\omega }}_{d}/{\omega }_{r}=1.7$, *A* = 0.215, $\tilde{b}=0.6$ and *m* = 2.0).

The temperature dependence of the bipartite entanglement has been numerically investigated, for the case of three oscillators, and reported in Fig. 5 where we plot ${E}_{N}^{\mathrm{1|23}}$ as function of *τ* for different values of $\tilde{T}$ for a fixed value of $\tilde{b}\mathrm{=0.6}$. From this figure we can state that a changing temperature does not affect the entanglement rate but it only slightly modify the initial entanglement time as in the case of two oscillators. We also observe that for a fixed value of *τ* the entanglement decreases as temperature increases.

**Figure 6:**
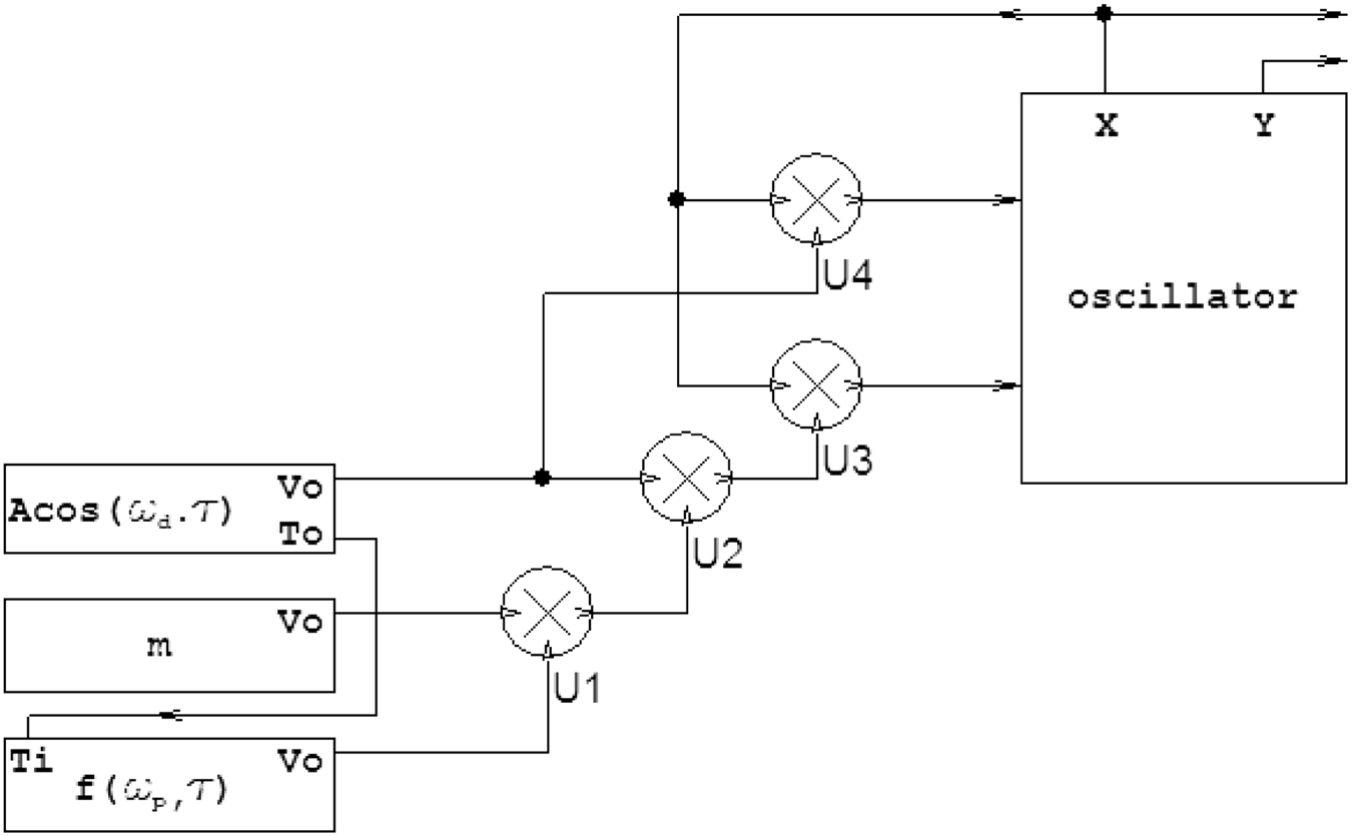
Electronic block scheme. Scheme of the analog implementation of the perturbed Mathieu’s oscillator according to eq. (17).

## Conclusions

In this work we have considered the bipartite quantum entanglement for a system of three oscillators in contact with a thermal reservoir at high temperature. The analysis of the classical counterpart by means of a suitable control implemented on a electronic single parametric oscillator allows to know the regions where entanglement will be originated. Peculiar differences emerge when the systems with two and three oscillators are compared, as the reduction of entanglement related with monogamy and the opposite behaviour in their initial times. We also note that the average bipartite entanglement rate is approximately a linear function of *μ* and it presents a bifurcation, related to the coupling between the two oscillators.

Although, our experimental measurements are limited to the classical system we believe that future experiments confirming the quantum features could be done by using coupled optomechanical cavities as recently pointed out in refs 17–19.

## Methods

The perturbed Mathieu oscillator, described by equation (17), has been implemented by an analog electronic circuit schematically represented in Fig. 5. The oscillator block has been realized employing four commercial operational amplifiers embedded in one LT1114CN chip (by *Linear Technology*). A function generator Hameg HM 8131-2 provides the sinusoidal driving signal $Acos({\tilde{\omega }}_{d}\tau )$ and a multiplier chip MLT04G (by *Analog Devices*) implements the product $Acos({\tilde{\omega }}_{d}\tau )\cdot x$ (multiplier *U*4 in the block scheme). A circuital branch, consisting of two function generators (Hameg HM 8131-2 and Tabor 8024) and some multipliers (MLT04G) (*U*1, *U*2 and *U*3 in the scheme), ensures the square waveform *m* · *f*(*ω*_*p*_, *t*) and the operation $m\cdot f({\omega }_{p},t)\cdot cos({\tilde{\omega }}_{d}\tau )\cdot x$. The perturbation is triggered by the signal $Acos({\tilde{\omega }}_{d}\tau )$ in such a way that the rising edge of the step signal is maintained in coincidence with the maximum of the sinusoidal driving signal $Acos({\tilde{\omega }}_{d}\tau )$. The experimental data have been acquired by means of a TDS 7104 Tektronix digital oscilloscope.

## Electronic supplementary material

Supplementary Information
