# Supplementary material for: Control of entanglement dynamics in a system of three coupled quantum oscillators
Source: Sci Rep. 2017 Aug 30;7:9957. doi: 10.1038/s41598-017-09989-2 (PMC5577133; doi:10.1038/s41598-017-09989-2)
Supplement: Supplementary file 1 — Supplementary Information [file 41598_2017_9989_MOESM1_ESM.pdf]

Control of entanglement dynamics in a system of three coupled quantum oscillators
Supplementary Information

J. C. Gonzalez-Henao,[1,2] E. Pugliese,[3] S. Euzzor,[3] R. Meucci,[3] J. A. Roversi,[1] and F. T. Arecchi[3,4]
[1]*Instituto de Física "Gleb Wataghin", Universidade Estadual de Campinas, Unicamp 13083-970, Campinas, São Paulo, Brazil*
[2]*Departamento de Física, Universidad de Sucre,Cra 28 No 5-267 Puerta Roja, Sincelejo, Sucre, Colombia*
[3]*Istituto Nazionale di Ottica, Consiglio Nazionale delle Ricerche, Largo E. Fermi 6, Firenze, Italy*
[4]*Emeritus of Physics, Università degli Studi di Firenze, Italy*

I. COUPLING BETWEEN QUANTIC OSCILLATIONS

In this section we provide details of the Hamiltonian diagonalization and the way to obtain the covariance matrix elements for the system of 3 oscillators in contact with the thermal reservoir.
In such a system, defined by Eqs. (1)-(3) [1], the Hamiltonian can be written as

$$H_T = H_S + H_{SR} \tag{1}$$

$$H_S = \frac{1}{2}\sum_{i=1}^{N}\left(\frac{P_i^2}{m_i} + m_i\omega^2(t)X_i^2\right) + \sum_{i>j=1}^{N} c(t)X_iX_j \tag{2}$$

$$H_{SR} = \sum_{k=1}^{\infty}\frac{p_k^2}{2m_k} + \frac{m_k\omega_k^2x_k^2}{2} - \sqrt{2}c_kx_k\left(\sum_{i=1}^{N}X_i\right) + \frac{c_k^2}{2m_k\omega_k^2}\left(\sum_{i=1}^{N}X_i\right)^2. \tag{3}$$

In order to diagonalize this Hamiltonian we introduce the transformation $[X] = R.[X']$ and $[P] = R.[P']$ based on the orthogonal matrix R:

$$R = \begin{pmatrix} \frac{1}{\sqrt{3}} & 0 & -\sqrt{\frac{2}{3}} \\ \frac{1}{\sqrt{3}} & \frac{1}{\sqrt{2}} & \frac{1}{\sqrt{6}} \\ \frac{1}{\sqrt{3}} & -\frac{1}{\sqrt{2}} & \frac{1}{\sqrt{6}} \end{pmatrix} \tag{4}$$

that transforms H_T, using the normalized operators X_i and P_i, in the following form [1]:

$$H'_T = H'_1 + H'_2 + H'_3 \tag{5}$$

$$H'_1 = \frac{1}{2}\left(\Omega_+^2(t)X_1'^2 + P_1'^2\right) + \sum_{k=1}^{\infty}\frac{p_k^2}{2} + \frac{\omega_k^2x_k^2}{2} - \sqrt{2}c_kx_kX'_1 + \frac{c_k^2}{2\omega_k^2}X_1'^2 \tag{6}$$

$$H'_2 = \frac{1}{2}\left(\Omega_-^2(t)X_2'^2 + P_2'^2\right) \tag{7}$$

$$H'_3 = \frac{1}{2}\left(\Omega_-^2(t)X_3'^2 + P_3'^2\right) \tag{8}$$

where $\Omega_+^2(t) = \left(\omega(t)^2 + 2c(t)\right)/\omega_0^2$ and $\Omega_-^2(t) = \left(\omega(t)^2 - c(t)\right)/\omega_0^2$. It is important to note that the operators X'_i and P'_i commute:

$$[X'_i, X'_j] = \left[\sum_{l=1}^{3}R_{il}X_l, \sum_{m=1}^{3}R_{jm}X_m\right] = \sum_{l,m=1}^{3}R_{il}R_{jm}[X_l, X_m] = 0$$

$$\left[P_i', P_j'\right] = \left[\sum_{l=1}^{3} R_{il}P_l, \sum_{m=1}^{3} R_{jm}P_m\right] = \sum_{l,m=1}^{3} R_{il}R_{jm}\left[P_l, P_m\right] = 0$$

$$\begin{aligned}\left[X_i', P_j'\right] &= \left[\sum_{l=1}^{3} R_{il}X_l, \sum_{m=1}^{3} R_{jm}P_m\right] = \sum_{l,m=1}^{3} R_{il}R_{jm}\left[X_l, P_m\right] \\ &= \sum_{l,m=1}^{3} R_{il}R_{jm}\delta_{lm} = \sum_{l=1}^{3} R_{il}R_{jl} = i\delta_{ij}\end{aligned}$$

because the matrix R is orthonormal.

II. INITIAL CONDITIONS

Using as initial condition the state $|\Psi(0)\rangle = |\alpha_1\rangle_1 \otimes |\alpha_2\rangle_2 \otimes |\alpha_3\rangle_3 = |\alpha_1, \alpha_2, \alpha_3\rangle$ where the states $|\alpha_i\rangle_i$ are coherent states, we have in the position representation (coordinate representation) $\{X_1, X_2, X_3\}$:

$$\begin{aligned}\langle X_1, X_2, X_3 |\Psi(0)\rangle &= \langle X_1, X_2, X_3 |\alpha_1, \alpha_2, \alpha_3\rangle \\ &= \exp\left(-\frac{1}{2}X_1^2\omega_0^2 - \frac{1}{2}X_2^2\omega_0^2 - \frac{1}{2}X_3^2\omega_0^2\right. \\ &+ \sqrt{2\omega_0}\left(\alpha_1X_1 + \alpha_2X_2 + \alpha_3X_3\right) \\ &- \left.\frac{|\alpha_1|^2}{2} - \frac{|\alpha_2|^2}{2} - \frac{|\alpha_3|^2}{2} - \frac{\alpha_1^2}{2} - \frac{\alpha_2^2}{2} - \frac{\alpha_3^2}{2}\right) \qquad (9)\end{aligned}$$

To write the initial condition in the new coordinate system we need to substitute in the first line of transformation (4) the elements X_i by X_i'. In the second line we can write $\alpha_1X_1 + \alpha_2X_2 + \alpha_3X_3 = [\alpha]^T . [X] = [\alpha]^T .R.[X']$, and defining $[\alpha'] = R^T.[\alpha]$, in the last line of the above equation we need only change α_i by α_i'. In this way we can see that a rotation in the coordinate space generates a rotation in the coordinate space of coherent states. After the transformation the initial state becomes:

$$\begin{aligned}\langle X_1', X_2', X_3' |\Psi(0)\rangle &= \langle X_1', X_2', X_3' |\alpha_1', \alpha_2', \alpha_3'\rangle \\ &= \left(\frac{\omega_0}{\pi}\right)^{3/2}\exp\left(-\frac{1}{2}X_1'^2\omega_0^2 - \frac{1}{2}X_2'^2\omega_0^2 - \frac{1}{2}X_3'^2\omega_0^2\right. \\ &+ \sqrt{2\omega_0}\left(\alpha_1'X_1' + \alpha_2'X_2' + \alpha_3'X_3'\right) \\ &- \left.\frac{|\alpha_1'|^2}{2} - \frac{|\alpha_2'|^2}{2} - \frac{|\alpha_3'|^2}{2} - \frac{\alpha_1'^2}{2} - \frac{\alpha_2'^2}{2} - \frac{\alpha_3'^2}{2}\right). \qquad (10)\end{aligned}$$

As it can be seen the state continues to be a separable state in the new coordinate system.

III. COVARIANCE MATRIX

Noting that the Hamiltonian (5) is bilinear, the evolution of a coherent initial state generates a Gaussian state, implying that all quantum correlations are contained in the Covariance Matrix (CM) defined by the elements:

$$\sigma_{\mathcal{R}_i\mathcal{R}_j} = \frac{1}{2}\langle \mathcal{R}_i\mathcal{R}_j + \mathcal{R}_j\mathcal{R}_i\rangle - \langle \mathcal{R}_i\rangle\langle \mathcal{R}_j\rangle \qquad (11)$$

where the vector $[\mathcal{R}] = (X_1, P_1, X_2, P_2, X_3, P_3)$.

In the previous section, for decoupling the oscillators, we have written the Hamiltonian (1) in the coordinates $\{X_i', P_i'\}$. Noting that the Hamiltonians H_i' commute, the elements $\sigma_{X_i'X_j'} = \sigma_{P_i'P_j'} = 0$ for $i \neq j$ and the elements $\sigma_{\mathcal{R}_i\mathcal{R}_j}$ become:

$$\begin{aligned} \sigma_{X_iX_j} &= \sum_{k=1}^{3} R_{ik}R_{jk}\sigma_{X_k'X_k'} && (12)\\ \sigma_{P_iP_j} &= \sum_{k=1}^{3} R_{ik}R_{jk}\sigma_{P_k'P_k'} && (13)\\ \sigma_{X_iP_j} &= \sum_{k=1}^{3} R_{ik}R_{jk}\sigma_{X_k'P_k'} && (14) \end{aligned}$$

where R_{ik} are the elements of the matrix (4).

A. Covariance Matrix for $t = 0$

As obtained in the section II the initial states of the harmonic oscillators in the coordinates $\{X_i', P_i'\}$ are coherent states, so in order to determine the covariance matrix at $t = 0$ we introduce

$$X_i' = \frac{1}{\sqrt{2\omega_0}}\left(\hat{\mathcal{A}}_i + \hat{\mathcal{A}}_i^\dagger\right) \quad \text{and} \quad P_i' = \frac{1}{\sqrt{2\omega_0}i}\left(\hat{\mathcal{A}}_i - \hat{\mathcal{A}}_i^\dagger\right) \qquad (15)$$

with $\hat{\mathcal{A}}_i\left|\alpha_i'\right\rangle = \alpha_i'\left|\alpha_i'\right\rangle$. From these definitions we obtain:

$$\begin{aligned} \sigma_{X_i'X_j'}(0) &= \frac{1}{2\omega_0}\delta_{i,j} && (16)\\ \sigma_{P_i'P_j'}(0) &= \frac{\omega_0}{2}\delta_{i,j} && (17)\\ \sigma_{X_iP_j}(0) &= 0. && (18) \end{aligned}$$

These are the CM elements that are necessary to manipulate the whole parametric oscillators system.

B. Determination of the Covariance Matrix elements

Noting that the transformed Hamiltonians commute, the CM elements can be obtained independently of each other. In the case of the Hamiltonians (7) and (8), for $i = 2, 3$, the determination of the CM elements can be obtained, in the Heisenberg representation, through the following system of coupled differential equations:

$$\begin{aligned} &\dot{\sigma}_{X_i'X_i'}(t) = 2\sigma_{X_i'P_i'}(t) && (19)\\ &\dot{\sigma}_{P_i'P_i'}(t) = -2\Omega_-^2(t)\,\sigma_{X_i'P_i'}(t) && (20)\\ &\dot{\sigma}_{X_i'P_i'}(t) = \sigma_{P_i'P_i'}(t) - 2\Omega_-^2(t)\,\sigma_{X_i'X_i'}(t). && (21) \end{aligned}$$

The solution of this system is given by:

$$\begin{aligned} \sigma_{X_i'X_i'}(t) &= \sigma_{X_i'X_i'}(0)\,\Theta_2^2(t) + \sigma_{P_i'P_i'}(0)\,\Theta_1^2(t) + \sigma_{X_i'P_i'}(0)\,\Theta_2(t)\,\Theta_1(t)\\ \sigma_{P_i'P_i'}(t) &= \sigma_{X_i'X_i'}(0)\,\dot{\Theta}_2^2(t) + \sigma_{P_i'P_i'}(0)\,\dot{\Theta}_1^2(t) + \sigma_{X_i'P_i'}(0)\,\dot{\Theta}_2(t)\,\dot{\Theta}_1(t)\\ \sigma_{X_i'P_i'}(t) &= \sigma_{X_i'X_i'}(0)\,\Theta_2(t)\,\dot{\Theta}_2(t) + \sigma_{P_i'P_i'}(0)\,\Theta_1(t)\,\dot{\Theta}_1(t)\\ &+ \sigma_{X_i'P_i'}(0)\left(\Theta_2(t)\,\dot{\Theta}_1(t) + \dot{\Theta}_2(t)\,\Theta_1(t)\right) && (22) \end{aligned}$$

where the functions $\Theta_1(t)$ and $\Theta_2(t)$ are the solutions of the differential equation (16)[1], with $\Theta_1(t)$ corresponding to the initial condition ($\Theta_1(0) = 0$, $\dot{\Theta}_1(0) = 1$) and $\Theta_2(t)$ having the initial condition ($\Theta_2(0) = 1$,$\dot{\Theta}_2(0) = 0$).

Considering that the Hamiltonian H_1' has a term of interaction with the reservoir, the best way to determine the elements $\sigma_{X_1'X_1'}(t)$, $\sigma_{P_1'P_1'}(t)$ and $\sigma_{X_1'P_1'}(t)$ is the use of the Feymann-Vernon theory. For this purpose it is necessary to obtain the density matrix of the system $\rho(\mathcal{X},\mathcal{Y},t)$ (eq.15 of the [1], that depends on the function $J(\mathcal{X},\mathcal{Y},\mathcal{X}',\mathcal{Y}',t)$. This term is given by:

$$\begin{aligned} J(\mathcal{X},\mathcal{Y},\mathcal{X}',\mathcal{Y}',t) =& \frac{1}{N(t)}\exp\left(i\left\{S(x_{cl})-S(y_{cl})-\gamma\int_o^t ds\,(x_{cl}(s)-y_{cl}(s))\left(\dot{x}_{cl}(s)+\dot{y}_{cl}(s)\right)\right\}\right) \\ &\times\exp\left(-\int_0^\tau\int_0^s dsd\tau\,(x_{cl}(s)-y_{cl}(s))\,K(s-\tau)\,(x_{cl}(\tau)-y_{cl}(\tau))\right). \\ K(u) =& \frac{2\gamma}{\pi}\int_0^\infty \omega\cosh\left(\frac{\hbar\omega}{2K_BT}\right)\cos(\omega u)\,d\omega \end{aligned} \tag{23}$$

where $N(t)$ is a normalization function and $K(u)$ is a kernel function that in the Markovian regime depends on the temperature of the reservoir T and the dissipation rate γ [2]. The action function $S(.)$ is then calculated from the Lagrangian:

$$L\left(x,\dot{x},y,\dot{y},,t\right)=\frac{\dot{x}^2}{2}-\frac{\Omega_+^2(t)}{2}x^2-\frac{\dot{y}^2}{2}+\frac{\Omega_+^2(t)}{2}y^2-\frac{\gamma}{2}\left(\dot{x}+\dot{y}\right)(x-y), \tag{24}$$

The functions $x_{cl}(t)$ e $y_{cl}(t)$ are the solutions of the Euler-Lagrange equations [3]. Thus using the Lagrangian (24) we have:

$$\begin{cases} \ddot{x}+\gamma\dot{y}+\Omega_+^2(t)\,x &= 0 \\ \ddot{y}+\gamma\dot{x}+\Omega_+^2(t)\,y &= 0. \end{cases} \tag{25}$$

The solution of this system of equations (25) can be determined by applying the transformations $\mathbb{X}_+=(x+y)\exp\left(\frac{\gamma}{2}t\right)$ and $\mathbb{X}_+'=(x-y)\exp\left(-\frac{\gamma}{2}t\right)$, from which we get:

$$\begin{cases} \ddot{\mathbb{X}}_+(t)+\left(\Omega_+^2(t)-\frac{\gamma^2}{4}\right)\mathbb{X}_+(t) &= 0 \\ \ddot{\mathbb{X}}_+'(t)+\left(\Omega_+^2(t)-\frac{\gamma^2}{4}\right)\mathbb{X}_+'(t) &= 0 \end{cases} \tag{26}$$

that is the equation (16) of the [1]. The initial conditions for $x_{cl}(t)$ and $y_{cl}(t)$ are given by $x_{cl}(0)=\mathcal{X}'$, $y_{cl}(0)=\mathcal{Y}'$, $x_{cl}(t_f)=\mathcal{X}$ and $y_{cl}(t_f)=\mathcal{Y}$.
Finally we have used the following equations:

$$\sigma_{X_1'X_1'}(t)=\int_{-\infty}^{\infty}X_1'^2\rho(X_1',X_1',t)\,dX_1'-\left(\int_{-\infty}^{\infty}X_1'\rho(X_1',X_1',t)\,dX_1'\right)^2 \tag{27}$$

$$\sigma_{P_1'P_1'}(t)=\int_{-\infty}^{\infty}\frac{d^2}{dX_1'^2}\rho(X_1',X_1',t)\,dX_1'-\left(\int_{-\infty}^{\infty}-i\frac{d}{dX_1'}\rho(X_1',X_1',t)\,dX_1'\right)^2 \tag{28}$$

$$\begin{aligned} \sigma_{X_1'P_1'}(t)=&\frac{1}{2}\int_{-\infty}^{\infty}\left(-iX_1'\frac{d}{dX_1'}\rho(X_1',X_1',t)-i\frac{d}{dX_1'}X_1'\rho(X_1',X_1',t)\right)dX_1' \\ &-\left(\int_{-\infty}^{\infty}X_1'\rho(X_1',X_1',t)\,dX_1'\right)\left(\int_{-\infty}^{\infty}-i\frac{d}{dX_1'}\rho(X_1',X_1',t)\,dX_1'\right) \end{aligned} \tag{29}$$

to obtain the covariant matrix elements $\sigma_{X_1'X_1'}(t)$, $\sigma_{P_1'P_1'}(t)$ and $\sigma_{X_1'P_1'}(t)$.

C. Covariance Matrix symplectic eigenvalues

We consider the following full symmetric 6×6 Covariance Matrix for a system of three oscillators:

$$\sigma=\begin{pmatrix}\sigma_{11} & \sigma_{12} & \sigma_{12}\\ \sigma_{12} & \sigma_{11} & \sigma_{12}\\ \sigma_{12} & \sigma_{12} & \sigma_{11}\end{pmatrix},\quad \sigma_{11}=\begin{pmatrix}\sigma_{X1X1} & \sigma_{X1P1}\\ \sigma_{X1P1} & \sigma_{P1P1}\end{pmatrix},\quad \sigma_{12}=\begin{pmatrix}\sigma_{X1X2} & \sigma_{X1P2}\\ \sigma_{P1X2} & \sigma_{P1P2}\end{pmatrix}$$

Defining the block matrix $\Lambda = \begin{pmatrix} \sigma_{11} & \sigma_{12} \\ \sigma_{12} & \sigma_{11} \end{pmatrix}$, it is possible to determine the transpose symplectic eigenvalue in term of the determinants of the 4 matrix above

$$\begin{cases} \Delta = 8\left(\text{Det}\left\{\sigma_{11}\right\} + \text{Det}\left\{\sigma_{12}\right\}\right) & \epsilon_{-} = \sqrt{\Delta^2 - 64\text{Det}\left\{\Lambda\right\}} \\ \Delta_{12} = 4\text{Det}\left\{\sigma_{11}\right\} + 16\text{Det}\left\{\sigma_{12}\right\} & z_{\pm} = \left(\epsilon_{+} \pm \epsilon_{-}\right)/\sqrt{4\text{Det}\left\{\sigma_{11}\right\}} \\ \Delta_T = 8\left(\text{Det}\left\{\sigma_{11}\right\} - \text{Det}\left\{\sigma_{12}\right\}\right) & \lambda = \left(\sqrt{4\text{Det}\left\{\sigma_{11}\right\}} - z_{+}\right)\left(\sqrt{4\text{Det}\left\{\sigma_{11}\right\}} - z_{-}\right) \\ \epsilon_{+} = \sqrt{\left(\Delta - 16\text{Det}\left\{\sigma_{11}\right\}\right)^2 - 64\text{Det}\left\{\Lambda\right\}} & \end{cases}$$

in this way the transpose symplectic eigenvalue $\tilde{n}_{-}$, introduced in the eq. (21) of the manuscript, assumes the form:

$$\tilde{n}_{-} = \sqrt{\frac{\Delta_T + \frac{16\text{Det}\{\Lambda\}}{\lambda^2} - \sqrt{\left(\Delta_T + \frac{16\text{Det}\{\Lambda\}}{\lambda^2}\right)^2 - \frac{256\text{Det}\{\sigma\}}{\lambda^2}}}{2}}$$

---

[1] J.C. Gonzalez-Henao, E. Pugliese, S. Euzzor, R. Meucci,J.A. Roversi and F.T. Arecchi, Experimental control of entanglement dynamics in a system of three coupled quantum oscillators.

[2] C. Zerbe, and P. Hänggi, Brownian parametric quantum oscillator with dissipation, *Phys. Rev. E* **52**, 1533 (1995).

[3] H. Goldstein and V. Twersky, Classical Mechanics, *Physics Today* **5**, 19 (1952).
